# Sphingosine Kinase-1 Is Central to Androgen-Regulated Prostate Cancer Growth and Survival

**DOI:** 10.1371/journal.pone.0008048

**Published:** 2009-11-26

**Authors:** Audrey Dayon, Leyre Brizuela, Claire Martin, Catherine Mazerolles, Nelly Pirot, Nicolas Doumerc, Leonor Nogueira, Muriel Golzio, Justin Teissié, Guy Serre, Pascal Rischmann, Bernard Malavaud, Olivier Cuvillier

**Affiliations:** 1 CNRS, Institut de Pharmacologie et de Biologie Structurale, Toulouse, France; 2 Université de Toulouse, UPS, IPBS, Toulouse, France; 3 CHU Toulouse, Hôpital Rangueil, Service d'Urologie et de Transplantation Rénale, Toulouse, France; 4 CHU Toulouse, Hôpital Rangueil, Laboratoire Anatomie Pathologique et Histologie-Cytologie, Toulouse, France; 5 CHU Toulouse, Hôpital Purpan, Laboratoire de Biologie Cellulaire et Cytologie, Toulouse, France; Bauer Research Foundation, United States of America

## Abstract

**Background:**

Sphingosine kinase-1 (SphK1) is an oncogenic lipid kinase notably involved in response to anticancer therapies in prostate cancer. Androgens regulate prostate cancer cell proliferation, and androgen deprivation therapy is the standard of care in the management of patients with advanced disease. Here, we explored the role of SphK1 in the regulation of androgen-dependent prostate cancer cell growth and survival.

**Methodology/Principal Findings:**

Short-term androgen removal induced a rapid and transient SphK1 inhibition associated with a reduced cell growth in vitro and in vivo, an event that was not observed in the hormono-insensitive PC-3 cells. Supporting the critical role of SphK1 inhibition in the rapid effect of androgen depletion, its overexpression could impair the cell growth decrease. Similarly, the addition of dihydrotestosterone (DHT) to androgen-deprived LNCaP cells re-established cell proliferation, through an androgen receptor/PI3K/Akt dependent stimulation of SphK1, and inhibition of SphK1 could markedly impede the effects of DHT. Conversely, long-term removal of androgen support in LNCaP and C4-2B cells resulted in a progressive increase in SphK1 expression and activity throughout the progression to androgen-independence state, which was characterized by the acquisition of a neuroendocrine (NE)-like cell phenotype. Importantly, inhibition of the PI3K/Akt pathway—by negatively impacting SphK1 activity—could prevent NE differentiation in both cell models, an event that could be mimicked by SphK1 inhibitors. Fascinatingly, the reversability of the NE phenotype by exposure to normal medium was linked with a pronounced inhibition of SphK1 activity.

**Conclusions/Significance:**

We report the first evidence that androgen deprivation induces a differential effect on SphK1 activity in hormone-sensitive prostate cancer cell models. These results also suggest that SphK1 activation upon chronic androgen deprivation may serve as a compensatory mechanism allowing prostate cancer cells to survive in androgen-depleted environment, giving support to its inhibition as a potential therapeutic strategy to delay/prevent the transition to androgen-independent prostate cancer.

## Introduction

Prostate cancer is the most frequent malignancy accounting for 25% of all newly diagnosed cancers in men and is the second leading cause of death from cancer [1]. Primary treatment with surgery or radiation therapy in patients with organ-confined prostate cancer demonstrates overall 10-year survival rates of over 75% [2], [3]. In spite of that, it is estimated that approximatively 15% of the patients present locally advanced or metastatic disease, and about 40% of patients will relapse after local therapy [4].

Prostate cancer cell proliferation is regulated by androgens and androgen deprivation therapy (ADT) is the standard of care in the management of patients with advanced disease. ADT is initially effective, reducing both prostate size and prostate-specific antigen (PSA) levels, but ultimately all patients become resistant to hormonal manipulation [4]. ADT induces changes in prostate cancer biology promoting its progression to the androgen-refractory state or hormone-refractory prostate cancer (HRPC) phenotype, with an associated life expectancy of only 15 to 20 months. It is not clear how prostate cancer cells make the transition from androgen-dependent to androgen-independent status after ADT. Among the multiple mechanisms involved in circumventing the effects of androgen ablation, the activation of the phosphatidylinositol-3-kinase/Akt (PI3K/Akt) signaling has been described as a central pathway [5], [6], [7], [8], [9]. Importantly, clinical studies have confirmed the importance of Akt activation in prostate cancer progression to androgen independence and poor clinical outcome [10], [11], [12], [13], [14].

Numerous studies have shown that, after long-term ADT, prostate cancer cells acquire a neuroendocrine (NE)-like phenotype leading to tumor populations enriched in NE cells. NE cells constitute a minor component of the normal prostate gland and secrete several neuropeptides that can induce mitogenic effects on adjacent cancer cells in androgen-depleted conditions [15]. Although NE cells have been described decades ago, their functional roles in prostate cancer progression have only recently received considerable attention. Neuroendocrine tumor and serum biomarkers are up-regulated following ADT in prostate cancer patients indicative of a poor prognosis [16], [17], [18], [19]. Consistent to clinical observations, androgen withdrawal-induced NE differentiation is also seen in cell culture and animal models [20], [21], [22], [23], [24], and the transgenic adenocarcinoma of the mouse prostate model of prostate (TRAMP) cancer shows a marked increase in prostate neuroendocrine cell population with disease progression [25].

Sphingosine 1-phosphate (S1P) is a lipid mediator which plays a major regulatory role in tumor cell growth, survival, invasion, and angiogenesis [26]. The balance between the cellular levels of S1P and its metabolic precursors ceramide and sphingosine is regarded as a switch that could determine whether a cell proliferates or undergoes apoptosis or growth arrest [27]. A key regulator of this balance is the sphingosine kinase-1 (SphK1), the enzyme converting sphingosine into S1P. SphK1 serves the dual function of producing the pro-growth, anti-apoptotic S1P, and decreasing intracellular levels of pro-apoptotic ceramide. Further supporting a role for SphK1 in promoting cancer, SphK1 has been found to act as an oncogene [28], its mRNA is overexpressed and positive immunostaining for SphK1 was found in various tumors [29], [30], [31], [32], [33], and the increase in SphK1 expression in tumor biopsies was correlated with short survival rate in patients with glioblastoma and breast cancers [30], [34]. In addition, SphK1 enzymatic activity and expression are markedly increased in tumor samples from prostate cancer patients (as compared with normal counterparts) correlating with other markers such as PSA level, tumor grade as well as with the clinical outcome after prostatectomy (Malavaud and Cuvillier, submitted). While SphK1 activity can be stimulated by a wide array of growth factors [26], we have previously shown in prostate cancer cell and animal models that anticancer treatments (chemotherapeutic agents or ionizing radiations) lead to its inhibition suggesting that SphK1 could act a sensor to anticancer therapies [35], [36], [37], [38].

In this study, we explored the potential role of SphK1 in the regulation of androgen-dependent cell growth and survival in the hormone-sensitive LNCaP prostate cancer cell model. For the first time, we show that androgen deprivation exerts a contrasting effect on SphK1. While short-term androgen withdrawal induced a temporary inhibition of SphK1, chronic androgen depletion triggered an up-regulation of SphK1 correlating with the NE differentiation of LNCaP and C4-2B cells supporting the involvement of SphK1 in the progression toward androgen-independence.

## Results

### Short-Term Androgen Deprivation Decreases Cell Proliferation in LNCaP - but Not in PC-3 Cells - and Is Associated with Decreased SphK1 Activity

**Figure 1:**
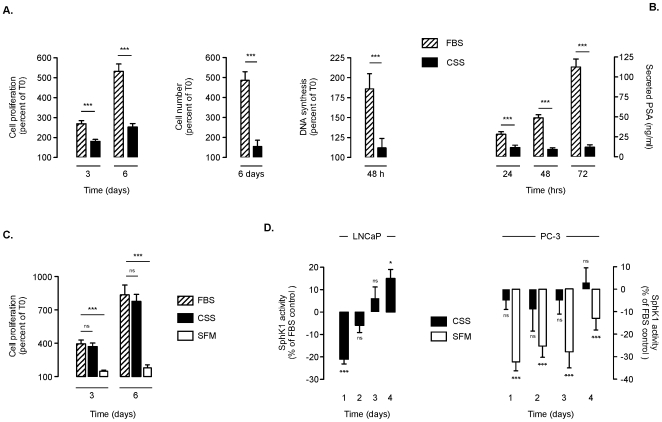
Effect of short-term androgen deprivation on cell growth and SphK1 activity. Overnight serum deprived LNCaP (***A***, ***D***) and PC-3 (***C***, ***D***) cells were incubated in presence of 5% FBS, 5% CSS or without serum (SFM) for the indicated times. Cell proliferation in LNCaP (***A***, ***left panel***) and PC-3 cells (***C***) was determined by MTT assay and expressed as percent of control at the beginning of the experiment (Day 0). Cell number (***A***, ***middle panel***) and DNA synthesis (***A***, ***right panel***) were measured as described in Materials and Methods. *Columns*, mean of at least twenty-four independent experiments for MTT assay and six experiments for cell counting and DNA synthesis; *bars*, SD. The *P* values between the means are as follow: ***, *P*<0.001. Secreted PSA level was measured in culture media from LNCaP cells (***B***). *Columns*, mean of at least six independent experiments; *bars*, SD. The *P* values between the means are as follow: ***, *P*<0.001. ***D***, SphK1 activity was determined in both LNCaP and PC-3 cells and expressed as percent of FBS-treated cells. *Columns*, mean of at least twelve and six independent experiments for LNCaP and PC-3 cells respectively; *bars*, SD. The *P* values between the means are as follow: ***, *P*<0.001; **, *P*<0.01; *, *P*<0.05; ns, non significant.

Cell proliferation was markedly reduced overtime in CSS (androgen-free medium)-treated LNCaP cells when compared to FBS (which contains low levels of androgens) treated cells (Fig. 1A, left panel). To support MTT results, cell counting (Fig. 1A, middle panel) and [^3^H]thymidine incorporation (Fig. 1A, right panel) measurements confirmed that CSS conditions had a dramatic effects on cell number and DNA synthesis establishing that MTT could be used as a surrogate index for cellular proliferation in our cell model. This was also correlated with the secretion of PSA whose level was strongly reduced in both CSS-treated cells as compared to FBS-treated cells (Fig. 1B). On the contrary, androgen deprivation did not alter the growth of PC-3 hormono-refractory (HR) cells whose growth was only altered by serum starvation (Fig. 1C).When compared to FBS conditions, androgen depletion in LNCaP induced a notable decrease in SphK1 activity within the first 24 h (Fig. 1D, left panel). Later, a rebound in SphK1 activity was observed, which became significant beyond 4 days of treatment (Fig. 1D, left panel). In PC-3 cells, a significant and lasting decrease in SphK1 activity was only observed under serum deprivation conditions (Fig. 1D, right panel). mirroring the impact on cell proliferation (Fig. 1C). As anticipated in PC-3 cells that are unaffected by CSS conditions, no significant SphK1 changes could be evidenced (Fig. 1C, D).

### The Efficacy of Castration in Orthotopically Xenotransplanted SCID Mice Is Associated with SphK1 Inhibition

**Figure 2:**
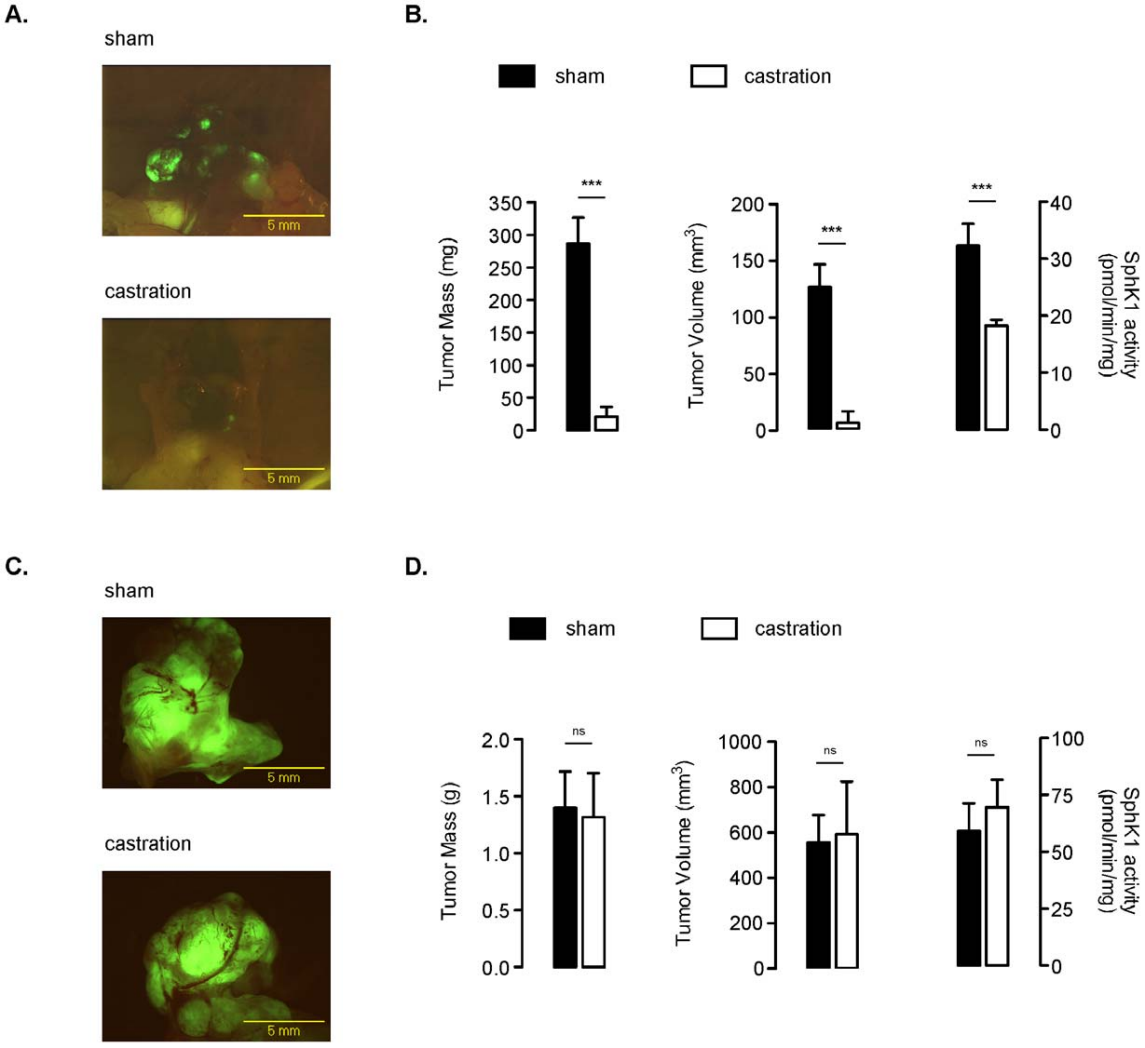
Effect of castration on tumor growth and SphK1 activity of established GFP-expressing human LNCaP and PC-3 cells in SCID mice. Nine and three weeks after SOI of LNCaP/GFP and PC-3/GFP cells respectively, SCID mice were randomized into two groups. These animals were then subjected to castration or sham treatment. Representative fluorescent primary LNCaP (***A***) and PC-3 (***C***) tumors from sham- and castration-treated animals at the time of autopsy (7 days post-treatment). Tumor mass of excised primary LNCaP (***B***, ***left panel***) and PC-3 (***D***, ***left panel***) GFP-labeled tumor. *Columns*, means from 8 animals; *bars*, SE. SphK1 activity was measured in tissue extracts obtained from sham-, and castration-treated LNCaP (***B***, ***right panel***) and PC-3 (***D***, ***right panel***) tumor bearing animals. *Columns*, means from 8 animals; *bars*, SE. The two-tailed *P* values between the means are as follow: ***, *P*<0.001; or ns, not significant.

The effect of androgen deprivation was next examined *in vivo* using a surgical orthotopic implantation (SOI) of LNCaP and PC-3 cells overexpressing GFP [36]. Nine and three weeks after SOI of LNCaP/GFP and PC-3/GFP cells respectively, SCID mice were randomized into two groups and subjected to castration or sham treatment. As shown by high-magnification microscopy of a representative primary LNCaP/GFP tumor, castration induced a dramatic decrease in tumor volume and mass (Fig. 2A and B) within 7 days of treatment compared to sham-treated animals. The efficacy of castration was associated with a significant decrease in SphK1 activity in tissue extracts (Fig. 2B, right panel). Additionally, the effect on primary tumor was paralleled by a marked reduction in metastasis dissemination in the castration-treated group (Table S1). As expected, castration did not effect tumor development in SCID mice xenotransplanted with PC-3/GFP cells (Fig. 2C). Tumor masses and volumes were similar in both sham-treated and castrated animals (Fig. 2D), and SphK1 activity (Fig. 2D, right panel) as well as metastasis dissemination (Table S1) were comparable in both groups.

### SphK1 Overexpression Renders LNCaP Cells Less Sensitive to Androgen Depletion

**Figure 3:**
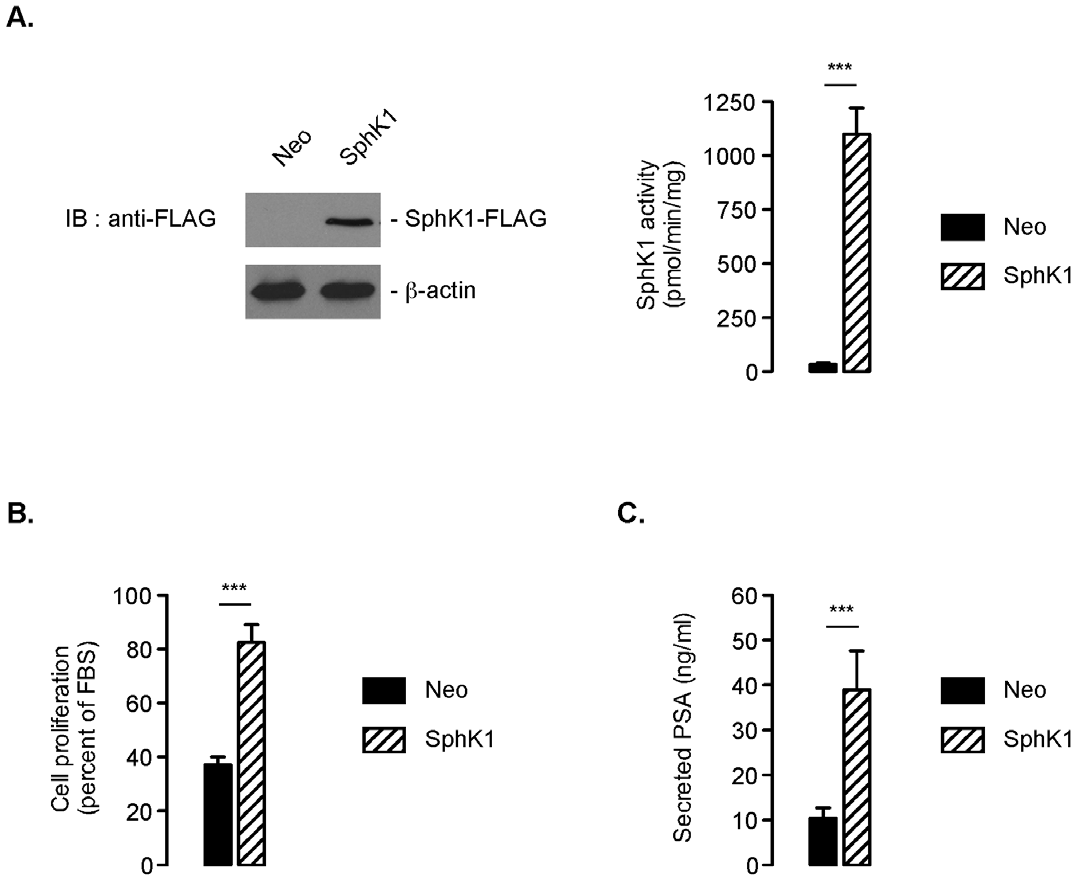
SphK1 overexpression renders LNCaP cells more resistant to short-term androgen deprivation. SphK1 expression in LNCaP cells was analyzed by Western blotting using anti-FLAG antibody (***A***, ***left panel***). Resting SphK1 activity was measured in LNCaP/neo and LNCaP/SphK1 cells (***A***, ***right panel***). ***B***, overnight serum deprived LNCaP cells were incubated in presence of 5% FBS or 5% CSS for 6 days. Cell proliferation was then determined and expressed as percent of 5% FBS-treated cells. *Columns*, mean of at least twelve independent experiments; *bars*, SD. The *P* values between the means are as follow: ***, *P*<0.001. ***C***, secreted PSA level was measured in culture media from LNCaP/Neo and LNCaP/SphK1 cells after 24 h of incubation in 5% CSS medium. *Columns*, mean of at least six independent experiments; *bars*, SD. The *P* values between the means are as follow: ***, *P*<0.001.

Because an inhibition of SphK1 was observed during short-term androgen deprivation *in vitro* (Fig. 1D) and *in vivo* (Fig. 2B), we verified whether transfection of LNCaP cells with SphK1 might render these cells more resistant to androgen depletion. Transfection efficiency was verified by immunoblotting (Fig. 3A, left panel). The SphK1 activity of SphK1-overexpressing LNCaP (Fig. 3A, right panel) was increased to ∼1100 pmol/mn/mg protein (i.e., ∼30-fold higher compared to that of empty-vector transfected cells). The instrumental role of SphK1 inhibition in reduced cell growth was confirmed by cell viability assays, which showed that LNCaP overexpressing SphK1 were markedly less sensitive to the effects of androgen depletion (Fig. 3B). Concomitantly, SphK1 enforced expression was associated with a higher secretion of PSA in CSS-treated LNCaP cells reflecting its effects on cell proliferation (Fig. 3C).

### Dihydrotestosterone Rapidly and Transiently Stimulates SphK1 Activity in an Androgen Receptor/PI3 Kinase-Dependent Manner

**Figure 4:**
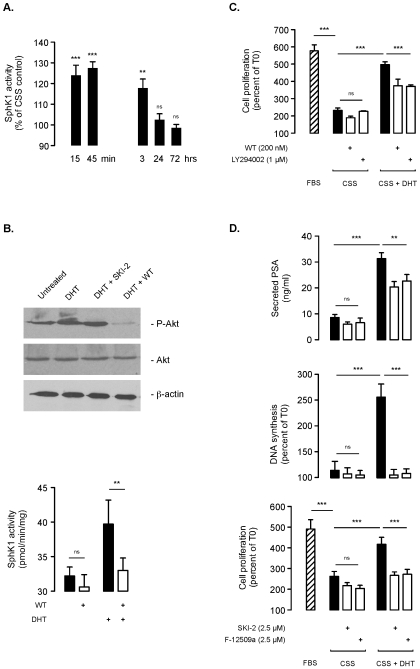
DHT stimulates SphK1 activity in a PI3 kinase-dependent manner in LNCaP cells. ***A***, overnight serum deprived LNCaP cells were incubated under 5% CSS conditions and treated with 10 nM DHT for the indicated times. SphK1 activity was quantified and expressed as percent of untreated cells. *Columns*, mean of at least six independent experiments; *bars*, SD. The *P* values between the means are as follow: ***, *P*<0.001; **, *P*<0.01; ns, non significant. ***B***, overnight serum deprived LNCaP cells were incubated with 10 nM DHT for 45 min in presence or not of 2.5 µM SKI-2 or 200 nM wortmannin (WT). Cell lysates were assayed for phospho-Akt and Akt expression by Western blot analysis. Similar results were obtained in three independent experiments (***top***). Overnight serum deprived LNCaP cells were incubated under 5% CSS conditions and treated with or without 10 nM DHT and 200 nM wortmannin (WT) for 45 min, and essayed for SphK1 activity (***bottom***). *Columns*, mean of at least five independent experiments; *bars*, SD. The *P* values between the means are as follow: **, *P*<0.01; ns, non significant. ***C***, overnight serum deprived LNCaP cells were incubated in FBS or in CSS conditions for 6 days with or without 200 nM wortmannin (WT) or 1 µM LY294002 in presence or not of 10 nM DHT as indicated. Cell proliferation was determined by MTT assay and expressed as percent of control at the beginning of the experiment (Day 0). *Columns*, mean of at least eight independent experiments; *bars*, SD. The *P* values between the means are as follow: ***, *P*<0.001; ns, non significant. ***D***, overnight serum deprived LNCaP cells were incubated in FBS or in CSS conditions with or without 2.5 µM of SKI-2 or F-12509a in presence or not of 10 nM DHT for 24 h (***top***), 48 h (***middle***) or 6 days (***bottom***). Secreted PSA level was quantified 24 h after the indicated treatment (***top***). DNA synthesis and MTT-based cell proliferation measurement were expressed as percent of control at the beginning of treatment, 2 and 6 days, respectively. *Columns*, mean of at least five independent experiments; *bars*, SD. The *P* values between the means are as follow: ***, *P*<0.001; **, *P*<0.01; or ns, non significant.

As a down-regulation of SphK1 activity was correlated with the removal of androgens in LNCaP cells (Fig. 1D), it was of interest to establish whether this could be reversed by the addition of androgens. Under CSS conditions, the addition of dihydrotestosterone (DHT) could trigger an early and transient stimulation of SphK1 (as early as 15 min) after which SphK1 activity returned to basal levels (Fig. 4A). The rapid stimulation of SphK1 was dependent on activation of the PI3K/Akt signaling, which has been shown to mediate the rapid effects of androgens [39], [40]. Indeed, while wortmannin (WT) inhibited both the activation of Akt (Fig. 4B, top) and SphK1 (Fig. 4B, bottom) induced by DHT, the SphK1 inhibitor SKI-2 did not impact Akt phosphorylation (Fig. 4B, top). The stimulation of the PI3K/Akt/SphK1 pathway was critical for transmitting the proliferative effects of DHT under CSS conditions. In fact, similar to the PI3K inhibitors WT and LY294002 (Fig. 4C), the SphK1 inhibitors SKI-2 and F-12509a could impede DHT-induced cell proliferation, DNA synthesis as well as PSA secretion (Fig. 4D).

**Figure 5:**
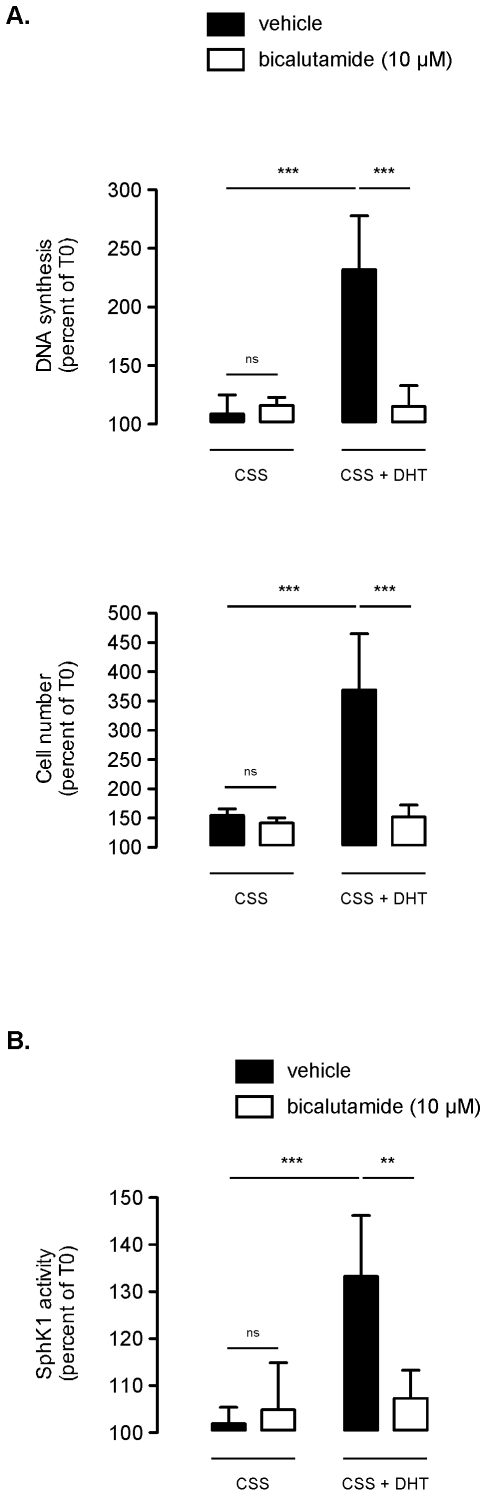
Bicalutamide inhibits cell proliferation and SphK1 stimulation triggered by DHT. Overnight serum deprived LNCaP cells were incubated under 5% CSS conditions and treated with or without 10 µM bicalutamide in presence or not of 10 nM DHT for 2 (***A***, ***top***) or 6 days (***A***, ***bottom***). DNA synthesis and cell counting measurements were expressed as percent of control at the beginning of treatment. ***B***, overnight serum deprived LNCaP cells were incubated with 10 nM DHT for 30 min in presence or not of 10 µM bicalutamide, and SphK1 activity was quantified and expressed as percent of control at the beginning of treatment. *Columns*, mean of at least five independent experiments; *bars*, SD. The *P* values between the means are as follow: ***, *P*<0.001; **, *P*<0.01; or ns, non significant.

The effect of androgen receptor antagonist was next examined with bicalutamide. As anticipated, LNCaP cell proliferation response at 10 nM DHT was totally blunted by 10 µM bicalutamide (Fig. 5A). The DHT-triggered stimulation of SphK1 activity was also completely inhibited in presence of androgen receptor antagonist (Fig. 5B).

### SphK1 Is Involved in Androgen Depletion-Induced Neuroendocrine Transdifferentiation of LNCaP and C4-2B Cells

**Figure 6:**
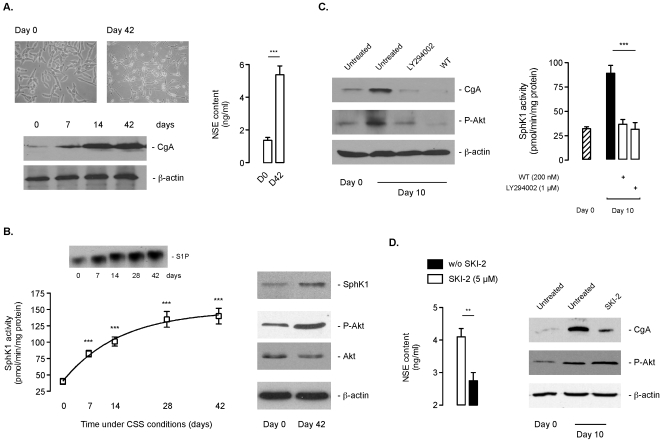
The progression of LNCaP to androgen-refractory state is associated with increase in SphK1 expression and activity. ***A***, representative phase-contrast images of LNCaP cells at the beginning of the experiment (Day 0) and after 42 days of incubation in charcoal stripped conditions (Day 42). Chromogranin A (CgA) content was evaluated by immunoblotting at the indicated times (***bottom***).Secreted Neuron Specific Enolase (NSE) level was measured in culture media from LNCaP cells at the indicated times (***right panel***). *Columns*, mean of five independent experiments; *bars*, SD. The two-tailed *P* values between the means are as follow: ***, *P*<0.001. SphK1 activity (***B***, ***left panel***) was determined at the indicated times in LNCaP cells incubated in CSS conditions. *Points*, mean of five independent experiments; *bars*, SD. The *P* values between the means are as follow: ***, *P*<0.001. ***Inset***, S1P content as shown by TLC. ***Right panel***, expression of SphK1, phospho-Akt, and Akt were analyzed by Western blotting at the indicated times. Similar results were obtained in five independent experiments. ***C***, expression of CgA and phospho-Akt (***left panel***) and SphK1 activity (***right panel***) were analyzed 10 days after treatment or not with 1 µM LY294002 or 200 nM wortmannin (WT) under charcoal stripped conditions. *Columns*, mean of five independent experiments; *bars*, SD. The two-tailed *P* values between the means are as follow: ***, *P*<0.001. ***D***, secreted NSE level was measured in culture media 10 days after treatment or not with 5 µM SKI-2 under charcoal stripped conditions (***left panel***). Expression of CgA and phospho-Akt were analyzed by western blot (***right panel***). *Columns*, mean of five independent experiments; *bars*, SD. The two-tailed *P* values between the means are as follow: **, *P*<0.01.

We next investigated the potential involvement of SphK1 in the transition to androgen refractory state after chronic androgen withdrawal. To this end, LNCaP and C4-2B cells, which have been previously reported to acquire a neuroendocrine (NE) phenotype [20], [21], [23], [41], [42], [43] were maintained under CSS conditions for up to 42 days. LNCaP cells exposed to a hormone-deficient medium underwent neuroendocrine (NE) morphological changes (apparent after approximatively 7–10 days) as indicated by soma compaction and development of long and branched neuritic extensions (Fig. 6A), whereas control LNCaP parental cells retained a fusiform epithelial morphology (Fig. 6A). In addition to morphological characteristics, transdifferentiated NE-like prostate cancer cells are defined by the expression of a number of neurosecretory products including chromogranin A (CgA) and neuron-specific enolase (NSE). CgA is considered to be the most reliable indicator of prostatic NE differentiation.

Consistent with previous observations [41], [44], immunoblots showed that CgA expression was very low in both parental LNCaP (Fig. 6A, lower panel) and C4-2B (Figure S1) cells, while its expression level was greatly enhanced over time in hormone-deficient medium. Furthermore, compared to non hormone-deprived LNCaP cells (D0), a 3-fold increase in NSE secretion to the medium was found after 42 days under CSS conditions indicative of a NE-like phenotype (Fig. 6A, right panel). Based on our previous observations that SphK1 activity upon androgen deprivation was only transiently inhibited with a significant rebound within 4 days of incubation in CSS medium (Fig. 1D), we then analyzed the SphK1 activity up to 42 days during the NE transdifferentiation process. Remarkably, SphK1 activity (Fig. 6B) increased progressively up to a 4-fold increase after 42 days of androgen depletion. This was further illustrated by a marked increase in S1P content (Fig. 6B, inset). In parallel, SphK1 protein expression was significantly increased (Fig. 6B, right panel). The activation of SphK1 was correlated with stimulation of Akt (Fig. 6B, right panel), a pro-survival signaling pathway reported to be up-regulated during NE transdifferentiation of LNCaP induced by androgen depletion [6], [7], [23], [45]. Comparable findings were observed in the C4-2B cell model (Figure S1).

In line with previous studies [6], pharmacological inhibition of the PI3K/Akt pathway could impede NE transdifferentiation of both LNCaP (Fig. 6C) and C4-2B (Figure S1) maintained for 10 days under CSS conditions. Of note, the blockade of CgA accumulation induced by the PI3K inhibitors WT and LY294002 was markedly associated with a decrease in both Akt phosphorylation and SphK1 activity (Fig. 6C, right panel and Figure S1).

To investigate the functional role of SphK1 in NE transdifferentiation, we examined the effect of the pharmacological inhibitor SKI-2 on LNCaP and C4-2B cells. In presence of SKI-2 (5 µM), the distinct NE-like morphology observed was essentially abolished with LNCaP exhibiting a rounded morphology with short rarely branched cellular processes (not shown). Moreover, both secretion of NSE (Fig. 6D, left panel) and CgA accumulation (Fig. 6D, right panel and Figure S1) were markedly diminished while Akt phosphorylation was unchanged hence suggesting that SphK1 inhibition can to a certain extent block the NE transdifferentiation.

**Figure 7:**
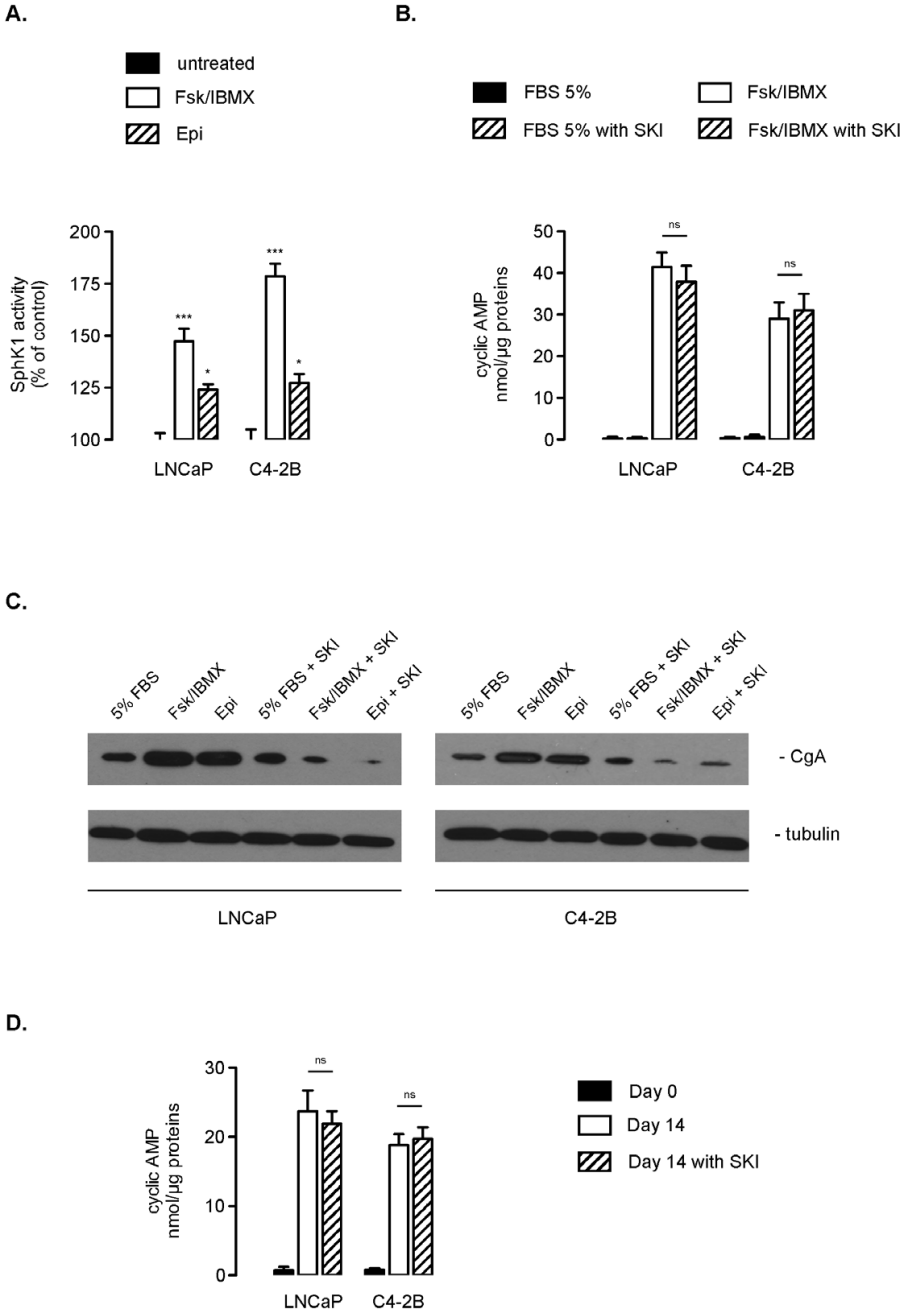
The increase in SphK1 activity during NED is cyclic AMP- dependent. ***A***, SphK1 activity was determined in LNCaP and C4-2B cells treated for 3 days in absence or in presence of 10 µM Fsk/10 mM IBMX or 10 µM Epi. ***B***, intracellular concentrations of cAMP were quantified in LNCaP and C4-2B cells treated for 3 days with 5% FBS or 10 µM Fsk/10 mM IBMX in absence or in presence of 5 µM SKI-2. ***C***, chromogranin A (CgA) content was evalated by immunoblotting in LNCaP and C4-2B cell extracts incubated for 3 days with or without 5 µM SKI-2 in presence of 5% FBS, 10 µM Fsk/10 mM IBMX or 10 µM Epi. Similar results were obtained in three independent experiments. ***D***, intracellular concentrations of cAMP were quantified in LNCaP and C4-2B cells before or 14 days after treatment or not with 5 µM SKI-2 under charcoal stripped conditions. *Columns*, mean of three independent experiments; *bars*, SD. The two-tailed *P* values between the means are as follow: ***, *P*<0.001; *, *P*<0.05; or ns, non significant.

Physiological or pharmacological agents that increase intracellular levels of cyclic AMP (cAMP) can induce the development of a NE morphology in LNCaP or C4-2 cells in presence of steroids [41], [44], [46]. Of note, cAMP increase has been previously reported to be associated with activation of SphK1 in osteoblasts [47]. Hence, we examined the role of cAMP as a potential upstream regulator of SphK1 during NED. LNCaP and C4-2B cells were stimulated with the adenylate cyclase activator forskolin (Fsk) and the phosphodiesterase inhibitor IBMX, or with epinephrine (Epi), a β-adrenergic receptor agonist. The treatment with Fsk/IBMX or Epi strongly stimulated SphK1 activity (Fig. 7A), and correlated with increased cAMP content (Fig. 7B) and acquisition of NE morphology (data not shown) characterized biochemically by CgA accumulation (Fig. 7C). Interestingly, the pharmacoligical inhibition of SphK1 did not have any effect on cAMP levels (Fig. 7B) whereas it did markedly inhibit NED (Fig. 7C). Remarkably, under androgen deprivation conditions, a similar increase in cAMP intracellular levels was observed, which could not be blocked by pharmacological inhibition of SphK1 (Fig. 7D). These results evoke a role for cAMP for the stimulation of SphK1 activity during NED.

**Figure 8:**
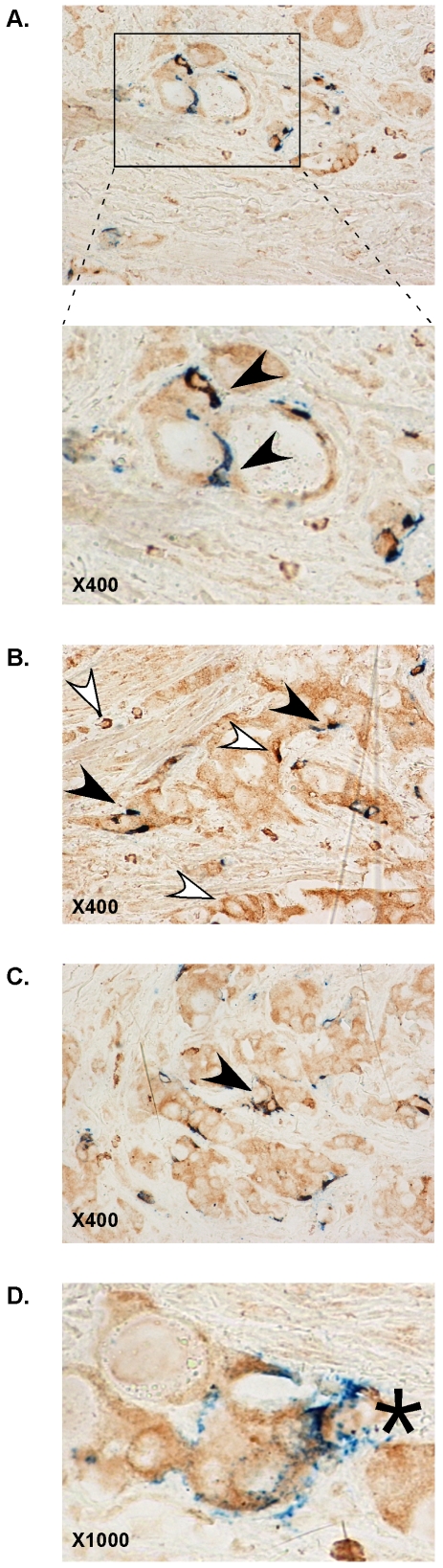
Co-expression of SphK1 and CgA in prostate cancer human samples. Dual immunostaining with anti-SphK1 (brown) and anti-CgA (blue) of a resection specimen harvested in a 84 y.o man with T4NxM1 prostate cancer under complete androgen blockade. Low serum PSA and positive serum NSE suggested poorly differentiated cancer with neuroendocrine features, as confirmed by pathological examination of the resected sample showing high grade prostate cancer (Gleason 9) with positive staining for CgA. Blue granular secretion characteristic of neuroendocrine differentiation is colocalized with brown SphK1 immune reactivity in neuron-like cells (solid arrowhead).

In order to determine whether the *in vitro* findings of the potential involvement of SphK1 in NE differentiation had any relevance to human disease, sections from a representative patient who underwent palliative transurethral resection for local recurrence under complete androgen blockade were immunostained for CgA and SphK1 expression (Fig. 8). SphK1 staining was restricted to cytoplasm with some cells being more intensely stained than the others (Fig. 8B, open arrowhead). Fibromuscular stroma did not stain positive for SphK1. CgA staining was observed in a minority of cancer cells, approximately 10%, in the form of secretory granuli (H, Fig. 8D). Co-expression of CgA in blue with brown SphK1 resulted in intense dark brown signal (solid arrowhead). Note in Fig. 8A (bottom) and Fig. 8C, the neuron-like appearance of the double-labeled cancer cell (solid arrow).

**Figure 9:**
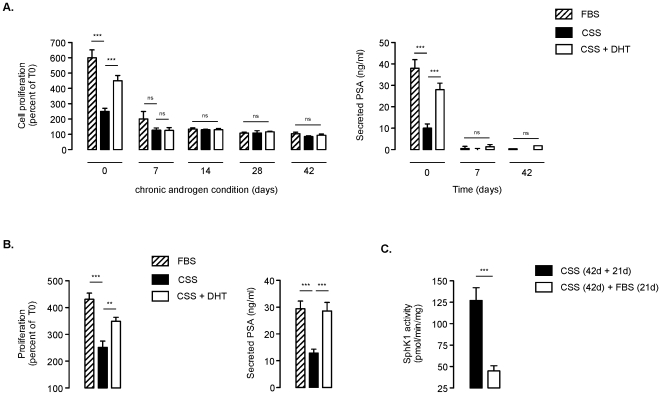
The reversability of the neuroendocrine-like phenotype by exposure to normal medium is associated with down-regulation of SphK1 activity. ***A***, LNCaP cells were left under CSS conditions for the indicated times (from 0 to 42 days). Cells were then collected and plated in CSS for 2 days to ensure complete adhesion before treatment with 5% FBS, 5% CSS with or without 10 nM DHT for 6 days (***left panel***) or 24 h (***right panel***). Cell proliferation was expressed as percent of control at the beginning of the 6 days treatment (***A***, ***left panel***). Secreted PSA level was quantified 24 h after treatment with 5% FBS, 5% CSS with or without 10 nM DHT of LNCaP cells (***A***, ***right panel***). *Columns*, mean of five independent experiments; *bars*, SD. The *P* values between the means are as follow: ***, *P*<0.001; or ns, not significant. ***B***, LNCaP cells were left in CSS conditions for 42 days and then cultured back for an additional 21 days in 5% FBS. Cells were then collected and plated in 5% FBS conditions for 2 days to ensure complete adhesion before treatment with 5% FBS, 5% CSS with or without 10 nM DHT for 6 days (***left panel***) or 24 h (***right panel***). Cell proliferation was expressed as percent of control at the beginning of the 6 days treatment (***B***, ***left panel***). Secreted PSA level was quantified 24 h after treatment with 5% FBS, 5% CSS with or without 10 nM DHT of LNCaP cells (***B***, ***right panel***). *Columns*, mean of five independent experiments; *bars*, SD. The *P* values between the means are as follow: ***, *P*<0.001; **, *P*<0.01; or ns, not significant. ***C***, LNCaP cells were cultured under CSS conditions for 42 days and then cultures for 21 days in 5% FBS or 5% CSS as indicated, then SphK1 activity was assessed. *Columns*, mean of five independent experiments; *bars*, SD. The two-tailed *P* values between the means are as follow: ***, *P*<0.001.

The transition to the androgen refractory state during chronic androgen depletion is characterized *in vitro* by a loss of response of androgen-deprived cells to either FBS or DHT [20]. As early as 7 days and by 14 days of androgen withdrawal, LNCaP were totally unresponsive after transfer to FBS conditions or when treated with DHT with regard to cell proliferation (Fig. 9A, left panel) or secretion of PSA (Fig. 9A, right panel). However, the NE-like phenotype is reversible upon replenishing the media with complete serum during several weeks [20]. When LNCaP androgen-depleted for 42 days were switched back to normal FBS medium, cell growth resumed over the period of the following week and, within the next 21 days in FBS medium, the cell morphology reverted to that of parental LNCaP cells (data not shown). As demonstrated in Fig. 8B, these cells were now not only able to grow normally and secrete PSA in presence of FBS like parental LNCaP (Fig. 1A), but also to respond again to the addition of DHT under CSS conditions similar to parental cells (Fig. 4C and D). Interestingly, the reversability induced by replenishing the media with complete serum for 21 days was associated with a profound down-regulation of SphK1 activity (Fig. 9C), which went back to the levels found in parental cells. These data suggest that SphK1 could represent an adaptable mechanism by which NE like-cells might survive in an environment deprived from androgens.

## Discussion

Compelling evidence has implicated SphK1 in promoting oncogenesis and response to anticancer therapies. On the one hand, SphK1 expression is up-regulated at the message or protein level in various solid tumors [29], [30], [31], [32], [33], and has been linked with poor survival for brain and breast cancers [30], [34]. On the other hand, anticancer treatments (chemotherapies, radiation therapy) trigger down-regulation of SphK1 activity in various cancer cell and animal models suggesting that SphK1 could act as a “sensor” to anticancer therapies particularly in prostate cancer [36], [37].

Here, we explored the potential role of SphK1 in the androgen-controlled cell growth and survival of the hormone-sensitive LNCaP prostate cancer cell model. As formerly established, androgen deprivation reduced cell growth and PSA secretion of LNCaP cells. Simultaneously, SphK1 activity was significantly down-regulated. The observed SphK1 inhibition was not relying on *de novo* synthesis of ceramide (data not shown), which has been previously reported to accumulate following androgen ablation in LNCaP cells [48]. Moreover, neither oxidative stress nor cathepsins - which have been previously implicated in SphK1 activity inhibition caused by other stimuli in various cell systems [49], [50] - seemed to be involved (data not shown). The short-term efficacy of androgen depletion was confirmed *in vivo* using an orthotopic model of xenotransplanted LNCaP cells where castration could not only noticeably reduce tumor size and metastasis dissemination but also induce a significant SphK1 inhibition. Of note, in hormono-insensitive PC-3 cells, neither androgen deprivation *in vitro* nor castration *in vivo* had any effect on tumor growth and SphK1 activity. SphK1 overexpression markedly inhibited the antiproliferative effect resulting from androgen removal. These results emphasize other findings establishing that enforced expression of SphK1 displays a cytoprotective effect against various stimuli such as chemotherapeutics notably in prostate cancer cells [36].

On the other hand, restoring the androgen environment was associated with a surge in SphK1 activity. The addition of dihydrotestosterone (DHT) induced a rapid and ephemeral activation of SphK1 suggestive of a non-genomic effect. The PI3K/Akt cascade has also been reported to participate in the rapid effects of DHT through a direct interaction between a membrane associated androgen receptor and PI3K [39], [40]. Herein, we demonstrate that SphK1 stimulation occurs downstream of the PI3K/Akt signaling since the canonical PI3K inhibitor, wortmannin could totally abrogate SphK1 stimulation upon DHT addition while SphK1 inhibition had no effect on DHT-induced phosphorylation of Akt. Further supporting the instrumental role played by SphK1 in the adaptation to hormonal environment, the pharmacological SphK1 inhibitors could potently (although not completely) inhibit the increase in cell proliferation and PSA secretion triggered by DHT.

Differential mechanisms are observed between short- and long-term androgen deprivation. It is well established both in cell culture and from clinical studies that the transition to the hormone-refractory state is correlated by the acquisition of a neuro-endocrine (NE) like phenotype [16], [17], [18], [19], [20], [21], [22], [23], [24], in which the activation of the PI3K/Akt signaling has been implicated [5], [6], [7], [8], [9], [10], [11], [12], [13], [14].

A major finding of our study is the spectacular increase in SphK1 activity, which appears to be dependent on cyclic AMP production, during the NE-differentiation process. It is tempting to speculate that the SphK1 activation upon chronic androgen deprivation may serve as an adaptive mechanism allowing prostate cancer cells to survive in androgen-depleted environment, as SphK1 is a well described pro-survival pathway *via* the effects of sphingosine 1-phosphate [27]. Consistent with an hypothesis of an adaptive functionality for SphK1, replenishing the media with complete serum was associated with the reversal of SphK1 activity towards its basal levels. In addition, pharmacological inhibition of SphK1 during chronic androgen depletion was capable of reversing at least partially the NE differentiation process. Further supporting this uncovered role for SphK1 during the transition to the hormone-refractory state, immunohistochemical studies of prostate cancer human samples showed for the first time that CgA positive-NE cells co-expressed SphK1. As NE cells secrete several neuropeptides with mitogenic effects on adjacent cancer cells in androgen-depleted conditions [15], it would be interesting to determine whether NE cells might represent a source of sphingosine 1-phosphate, which then could act in a paracrine manner to induce proliferation of epithelial cancer cells and angiogenesis [26].

Although effective, taxane-based chemotherapy for HRPC can delay the fatal outcome by only a few weeks or months, emphasizing the urgent need for new concepts to tackle the hormone-refractory state. By virtue of the uncovered role of SphK1 in mediating the transition from androgen-dependent to androgen refractory state, the pharmacological inhibition of SphK1 and its downstream signaling could represent a viable strategy to prevent or delay the progression to hormone-refractory prostate cancer.

## Materials and Methods

### Ethics Statement

All animal experiments were conducted as per the guidelines of the European Council Directive 86/609/EEC and approved by the local Inserm Animal Care and Use Committee.

### Cell Lines and Culture Conditions

Human prostate cancer hormone-sensitive LNCaP (DSMZ, Braunschweig, Germany) and C4-2B (Viromed, Minnetonka, MN), and hormone-insensitive PC-3 cells were cultured in RPMI 1640 containing 5% fetal bovine serum (FBS) and passaged less than 15–20 times. FLAG-tagged wild-type human SphK1 cDNA [51] was used for stable transfection of LNCaP cells. Mass pools of stable transfectants were selected in growth medium with 0.8 mg/ml G418. Empty vector and wild-type SphK1-transfected cells were designated LNCaP/Neo and LNCaP/SphK1, respectively. Stably transfected green fluorescent protein (GFP)-tagged LNCaP and PC-3 cells used for *in vivo* experiments were maintained in medium containing 0.5 mg/ml G418.

For optimal attachment, cells were plated and cultured for 48 h. Thereafter, the medium was replaced by serum-free and phenol red-free medium overnight before the indicated treatments.

### Materials

Culture medium, serum, and antibiotics were from Invitrogen (Cergy-Pontoise, France). Charcoal-stripped serum (CSS) was from Hyclone (Perbio Science, Brebières, France). Poly-d-Lysine was from BD Biosciences (Le Pont-De-Claix, France). Dihydrotestosterone (DHT), bicalutamide, forskolin (Fsk), isobutylmethylxanthine (IBMX), epinephrine (Epi) were from Sigma. The sesquiterpene quinone F-12509a [52], [53] was a gift from Dr Kohama (Daiichi-Sankyo Ltd., Tokyo, Japan). SKI-2 sphingosine kinase inhibitor [29], [36] and the PI3Kinase inhibitors, wortmannin and LY294002 were from Calbiochem (Fontenay-sous-Bois, France). [γ-^32^P]-ATP (3,000 mCi/mmol) was purchased from Perkin-Elmer (Courtaboeuf, France), and silica gel 60 high-performance TLC plates were from VWR (Fontenay-sous-Bois, France).

### Cell Viability Assay

Cell viability was carried out using the MTT (3-(4,5-dimethylthiazol-2-yl)-2,5 diphenyltetrazolium bromide) dye reduction assay [54]. Cells were seeded in poly-d-Lysine coated tissue culture plates (6-well) at a density of 36,000 and 14,000 cells/well for LNCaP and PC-3 cells respectively. At the end of the experiments, LNCaP and PC-3 cells were incubated with MTT reagent for 1 and 3 h, respectively.

### Cell Counting

At the indicated times, cells were harvested by trypsinization and resuspended in culture medium. Aliquots were diluted 50-fold in Isoton II (Coulter Corp., Miami, FL), and 200 µl duplicates were counted in a model Z1 Coulter particle counter and averaged.

### Measurement of DNA Synthesis

At the indicated times, cultures were pulsed with 1 µCi of [^3^H]-thymidine for 6 h and radioactivity incorporated into trichloroacetic acid-insoluble material measured as previously described [55].

### Western Blot Analysis and Antibodies

Western blotting was performed as previously described [54]. Rabbit anti-SphK1 (gift from Dr Pitson, IMVS, Adelaide, Australia), rabbit anti-Akt (Cell Signaling Technology # 9272), rabbit anti-Akt/phospho Ser473 (Cell Signaling Technology # 9271), mouse anti-CgA (Ventana Medical Systems), and mouse anti-FLAG (Sigma, clone M2) were used as primary antibodies. Proteins were visualized by ECL detection system (Perbio Science) using anti-rabbit or anti-mouse HRP-conjugated IgG (Bio-Rad, Marnes La Coquette, France). Equal loading was confirmed by probing the blots with the mouse anti-β-actin (Sigma, clone AC40) or anti-tubulin (Sigma, clone DM1A).

### Sphingosine Kinase-1 Assay

SphK1 activity was quantified as described previously [52], and determined in the presence of 50 µM sphingosine, 0.25% Triton X-100 and [γ-32P]-ATP (10 µCi, 1 mM) containing 10 mM MgCl_2_. The labelled S1P was separated by thin layer chromatography on silica gel 60 with 1-butanol/ethanol/acetic acid/water (80∶20∶10∶10, v/v) and visualized by autoradiography. Activity was expressed as picomoles of S1P formed/min/mg of protein.

### Quantitation of Secreted PSA and NSE Levels

Cells were seeded at an initial density of 7,800 and 4,200 cells/cm^2^ for LNCaP and PC-3 respectively. After treatment, culture media were collected and centrifuged. Total PSA was quantified by the Abbott MEIA PSA assay (Abbott AxSYM system® PSA total). NSE levels were determined by the TRACE (Time Resolved Amplified Cryptate Emission) technology, based on a non-radiative transfer of energy (Brahms NSE Kryptor®).

### cAMP Assay

The intracellular concentration of cAMP was carried out with the cAMP complete enzyme immunoassay kit from Assay designs (AnnArbor, MI, USA) following the recommendations of the manufacturer.

### Animals

6-week-old male SCID mice were obtained from Charles River (Saint Germain sur l'Arbresle, France).

### Surgical Orthotopic Implantation and Castration

Intraprostatic prostate cancer xenografts were established in SCID mice by surgical orthotopic implantation [36]. Mice were anesthetized by isoflurane inhalation and placed in the supine position. A lower midline abdominal incision was made and 20 µL tumor cell suspension (1×10^6^ cells) was injected into the dorsal lobe of the prostate. The incision was closed in two layers with 4/0 Dexon interrupted sutures. All procedures were done with a dissecting microscope. After anesthesia by isoflurane inhalation, testes were gently pushed into the scrotum and a 5 mm incision was made under. Each testis was surgically removed, and the spermatic cord and vascular plexus were tied with sterile suture to prevent hemorrhage. In sham controls, scrotal incision alone was performed.

### Autopsy, Histology and In Vivo Fluorescence Imaging

One week after castration, all mice were anesthetized and euthanized by cervical dislocation for direct internal imaging. A long midline incision was made to access the abdominal and the thoracic cavities. The fluorescent primary tumor was removed en bloc with the seminal vesicles and a meticulous fluorescence-assisted exploration was conducted to establish the presence of periaortic nodal extension, as well as adrenal, liver, and lung metastasis. GFP fluorescence was detected with a Leica MZFL III fluorescence stereomicroscope (Leica Microsystems, Wetzlar, Germany). High-resolution 16-bit images (1.392×1.040 pixels) were captured by a thermoelectrically cooled charge-coupled device camera (CoolSNAP HQ, Roper Scientific, Evry, France). To visualize the whole tumor and lymph nodes or micrometastases, ×8 and×35 magnifications were used, respectively. Selective excitation was produced with a Mercury Arc Lamp (HBO, Osram, Munich, Germany) and a GFP filter (Leica). Color images were obtained using a Micro*color tunable RGB filter (CRI, Woburn, MA). The images were processed for contrast and brightness and the fluorescence was analysed with MetaVue 6.2 software (Princeton Instruments, Trenton, NJ). The fluorescent area of the tumors was defined as a region of interest (ROI). A manual definition was used to distinguish between the fluorescent tumor area from other dark tissues. The area (a) of a ROI and the small diameter (d) were used to assess tumor volume (v) using the formula v = a×d×2/3.

### Immunohistochemistry

Sections were incubated for 4 hrs with primary rabbit anti-SphK1 antibody (1∶200) and revealed by the EnVision+ peroxidase kit and diaminobenzidine (DAB) chromogen substrate (Dako, Glostrup, Denmark). In the second reaction, sections were incubated with primary anti-Chromogranin A (clone LK2H10, Ventana Medical Systems, Tucson, AZ) in a dedicated staining system (Ventana) and revealed with anti-mouse immunoglobulins and alkaline phosphatase-conjugated streptavidin. Alkaline phosphatase activity was then revealed with Fast Blue (BB salt; 10 mg/10 ml TBS 0.1M, pH 8.2).

### Statistical Analysis

The statistical significance of differences between the means was evaluated using the unpaired Student's t test or the one-way analysis of variance (ANOVA) test. The frequencies of metastasis between the two groups were compared using Fisher's exact test. All statistical tests were two-sided and the level of significance was set at P<0.05. Calculations were performed using Instat (Graphpad Software, San Diego, CA).

## Supporting Information

Figure S1 The progression of C4-2B to androgen-refractory state is associated with increase in SphK1 expression and activity. A, Chromogranin A (CgA) content was evaluated by immunoblotting at the indicated times. B, SphK1 activity (left panel) was determined at the indicated times in C4-2B cells incubated in CSS conditions. Points, mean of five independent experiments; bars, SD. The P values between the means are as follow: ***, P<0.001. Expression of SphK1, phospho-Akt, and Akt were analyzed by Western blotting at the indicated times. (right panel). Similar results were obtained in five independent experiments. C, expression of CgA and phospho-Akt (left panel) and SphK1 activity (right panel) were analyzed 10 days after treatment or not with 1 µM LY294002 or 200 nM wortmannin (WT) under charcoal stripped conditions. Columns, mean of five independent experiments; bars, SD. The two-tailed P values between the means are as follow: ***, P<0.001. D, Expression of CgA and phospho-Akt were analyzed by western blot. Columns, mean of five independent experiments; bars, SD. The two-tailed P values between the means are as follow: **, P<0.01.
(0.38 MB TIF)

Table S1 (0.05 MB DOC)
