# Supplementary figures and images for: Sphingosine Kinase-1 Is Central to Androgen-Regulated Prostate Cancer Growth and Survival
Source: PLoS One. 2009 Nov 26;4(11):e8048. doi: 10.1371/journal.pone.0008048 (PMC2779655; doi:10.1371/journal.pone.0008048)

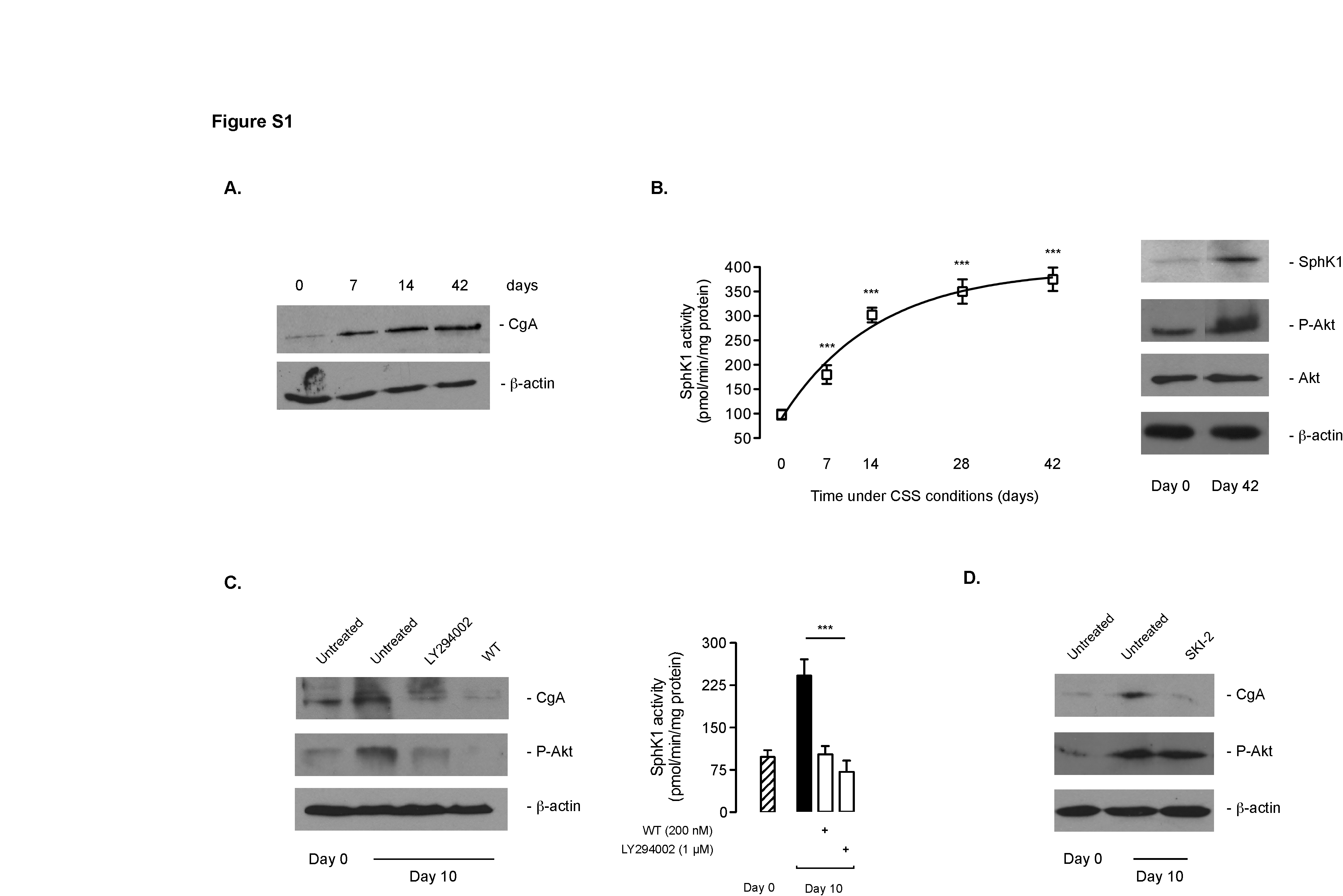

Supplement: Figure S1 — The progression of C4-2B to androgen-refractory state is associated with increase in SphK1 expression and activity. A, Chromogranin A (CgA) content was evaluated by immunoblotting at the indicated times. B, SphK1 activity (left panel) was determined at the indicated times in C4-2B cells incubated in CSS conditions. Points, mean of five independent experiments; bars, SD. The P values between the means are as follow: ***, P<0.001. Expression of SphK1, phospho-Akt, and Akt were analyzed by Western blotting at the indicated times. (right panel). Similar results were obtained in five independent experiments. C, expression of CgA and phospho-Akt (left panel) and SphK1 activity (right panel) were analyzed 10 days after treatment or not with 1 µM LY294002 or 200 nM wortmannin (WT) under charcoal stripped conditions. Columns, mean of five independent experiments; bars, SD. The two-tailed P values between the means are as follow: ***, P<0.001. D, Expression of CgA and phospho-Akt were analyzed by western blot. Columns, mean of five independent experiments; bars, SD. The two-tailed P values between the means are as follow: **, P<0.01. (0.38 MB TIF) [file pone.0008048.s001.tif]
