# Supplementary material for: Sphingosine Kinase-1 Is Central to Androgen-Regulated Prostate Cancer Growth and Survival
Source: PLoS One. 2009 Nov 26;4(11):e8048. doi: 10.1371/journal.pone.0008048 (PMC2779655; doi:10.1371/journal.pone.0008048)
Supplement: Table S1 — (0.05 MB DOC) [file pone.0008048.s002.doc]

## Table S1 : Pattern of metastatic dissemination

| Treatments |  | Sham  (LNCaP) | Castration  (LNCaP) |  | Sham  (PC-3) | Castration  (PC-3) |
| --- | --- | --- | --- | --- | --- | --- |
| No. of mice with metastases/  total No. of mice |  | 8/8 (100%) | 4/8 (50%) |  | 8/8 (100%) | 8/8 (100%) |
| Retroperitoneal lymph nodes |  |  |  |  |  |  |
| Periaortic lymph nodes |  | 1, 1, 1, 1, 1, 1, 1, 1 | 0, 1, 1, 0, 0, 1, 0, 1 |  | 1, 1, 1, 1, 1, 1, 1, 1 | 1, 1, 1, 1, 1, 1, 1, 1 |
| Periadrenal |  | 0, 1, 0, 1, 1, 1, 0, 0 | 0, 1, 0, 0, 0, 1, 0, 0 |  | 1, 1, 1, 1, 1, 1, 1, 1 | 1, 1, 1, 1, 1, 1, 1, 1 |
| Total No. of metastases |  | 12/16 | 6/16 **^§^** |  | 16/16 | 16/16 |
| Solid organs |  |  |  |  |  |  |
|  |  |  |  |  |  |  |
| Liver |  | 0, 0, 0, 0, 0, 0, 0, 0 | 0, 0, 0, 0, 0, 0, 0, 0 |  | 1, 1, 1, 1, 1, 1, 1, 1 | 1, 0, 1, 1, 1, 1, 1, 1 |
| Mesenteric |  | 0, 0, 0, 0, 0, 0, 0, 0 | 0, 0, 0, 0, 0, 0, 0, 0 |  | 0, 0, 0, 0, 0, 0, 0, 0 | 1, 0, 1, 0, 1, 1, 1, 0 |
| Pancreas |  | 0, 0, 0, 0, 0, 0, 0, 0 | 0, 0, 0, 0, 0, 0, 0, 0 |  | 1, 1, 1, 1, 1, 1, 1, 1 | 1, 0, 0, 1, 1, 1, 1, 1 |
| Lung |  | 0, 0, 0, 0, 0, 0, 0, 0 | 0, 0, 0, 0, 0, 0, 0, 0 |  | 1, 1, 1, 1, 1, 1, 1, 1 | 1, 0, 1, 1, 1, 1, 1, 1 |
| Total No. of metastases |  | 0/32 | 0/32 |  | 24/32 | 25/32 |

**^§^**  *P* = 0.0366 compared with sham-treated (Fisher’s exact test).
